# What do people benefit from a citizen science programme? Evidence from a Rwandan citizen science programme on malaria control

**DOI:** 10.1186/s12936-020-03349-8

**Published:** 2020-08-06

**Authors:** Domina Asingizwe, P. Marijn Poortvliet, Arnold J. H. van Vliet, Constantianus J. M. Koenraadt, Chantal M. Ingabire, Leon Mutesa, Cees Leeuwis

**Affiliations:** 1 College of Medicine and Health Sciences, University of Rwanda, Kigali, Rwanda; 2 Strategic Communication Group, Wageningen University, Wageningen, The Netherlands; 3 Environmental Systems Analysis Group, Wageningen University, Wageningen, The Netherlands; 4 Laboratory of Entomology, Wageningen University, Wageningen, The Netherlands; 5 Knowledge, Technology and Innovation Group, Wageningen University, Wageningen, The Netherlands

**Keywords:** Malaria, Citizen science, Perceptions, Benefits, Behaviour change, Social interaction, Collective action, Diffusion

## Abstract

**Background:**

Malaria control remains a challenge globally and in malaria-endemic countries in particular. In Rwanda, a citizen science programme has been set up to improve malaria control. Citizens are involved in collecting mosquito species and reporting mosquito nuisance. This study assessed what people benefit from such a citizen science programme. The analysis was conducted on how the citizen science programme influenced perceptions and behaviour related to malaria control.

**Methods:**

This study employed a mixed-methods approach using dissemination workshops, a survey, and village meetings as the main data collection methods. Dissemination workshops and village meetings involved 112 volunteers of the citizen science programme and were conducted to explore: (1) the benefits of being involved in the programme and (2) different ways used to share malaria-related information to non-volunteers. The survey involved 328 people (110 volunteers and 218 non-volunteers) and was used to compare differences in malaria-related perceptions and behaviour over time (between 2017 and 2019), as well as between volunteers and non-volunteers.

**Results:**

Malaria-related perceptions and behaviour changed significantly over time (between 2017 and 2019) and became favourable to malaria control. When the findings were compared between volunteers and non-volunteers, for perceptions, only perceived self-efficacy showed a significant difference between these two groups. However, volunteers showed significantly more social interaction, participation in malaria-related activities at the community level, and indoor residual spraying (IRS) acceptance. In addition, both volunteers and non-volunteers reported to have gained knowledge and skills about the use of malaria control measures in general, and mosquito species in particular among volunteers.

**Conclusion:**

The reported knowledge and skills gained among non-volunteers indicate a diffusion of the citizen science programme-related information in the community. Thus, the citizen science programme has the potential to provide individual and collective benefits to volunteers and society at large.

## Background

Despite major efforts to control the disease, malaria is still a severe health concern worldwide and particularly in Africa [1]. In Rwanda, the malaria indicator survey report conducted in 2017 indicated that malaria remains a burden where its prevalence is 7% in the general population and even higher (11%) among children aged between 5 and 14 years [2]. Furthermore, this prevalence rises to 17% in the general population in the Eastern province which is considered a malaria-endemic area [2]. Stalling malaria reduction and the reported malaria resurgence in some African countries, including Rwanda, hinder the progress towards malaria elimination.

The levels of investment in malaria control, access to, and acceptance of malaria control measures are still inadequate [1]. For example, a malaria indicator report conducted in Rwanda revealed that 72% of the visited households had access to long-lasting insecticidal nets (LLINs), and only 64% slept under LLINs the night before the survey [2]. To improve the uptake of malaria control measures, a comprehensive approach that operates at local levels, and that engages affected community members is encouraged by the World Health Organization [1, 3]. Furthermore, citizens’ engagement in malaria prevention activities has been proposed to improve the consistent use of malaria control measures [4]. However, how and to what extent such engagement may affect malaria-related perception and behaviour is still underexplored.

Citizen science, defined here as the engagement of citizens in scientific research, has been implemented in different disciplines [5–8]. Especially in the field of biology, many citizen science projects have been established [9]. In the context of the research programme Environmental Virtual Observatories for Connective Action (EVOCA) [10], with the ultimate goal of malaria control through improving the consistent use of malaria control measures, a Citizen Science Programme (CSP) for malaria control in Rwanda’s Ruhuha sector was set up [11]. This CSP for malaria control sought to increase insight into the spatial and temporal variation in (malaria) mosquito populations, mosquito nuisance, and confirmed malaria cases, in a rural area where this type of information was not readily available. Volunteers were asked to report these variables. This CSP was co-designed with citizens through participatory design workshops conducted in August 2018 [11]. Throughout reporting of citizen science data, monthly feedback through Short Message Service (SMS) and quarterly dissemination workshops were provided to the volunteers [11].

Citizen science has the potential to generate large quantities of data and engage citizens to better address and respond to complex environmental and societal issues, thereby enhancing the health and wellbeing of the population [12, 13]. This engagement improves citizens’ knowledge, as well as perceptions and behaviour [12]. As volunteers continue their participation in citizen science, they may expand their social networks [14, 15]. The network can either be within the volunteer group or beyond and thus may involve non-volunteers (those who do not submit citizen science data). Through the interaction between volunteers and non-volunteers, the impact of a citizen science project can be transferred and diffuse to other community members [16, 17]. The non-volunteers can be influenced by activities of the project (including the feedback or results provided by scientists) [17, 18], or by the discussion and interaction initiated by the volunteers [17]. Interaction and sharing of experiences from participation can increase social capital (here referred to as fostering the network of community members to improve malaria control) [14]. Meeting and interacting with other community members can increase openness and trust as well [12, 14].

Despite its potential, there is a lack of evidence for the impact of engaging people in a citizen science programme on both volunteers and non-volunteers, and to what extent and how it stimulates the consistent use of malaria control measures and participation in malaria control activities. Particularly, it is unclear to what extent malaria-related perceptions and behaviour change over time, and how they differ between those who directly contribute to the citizen science data and those who do not. To address this gap, this paper intends to explore the effect of the CSP for malaria control in Rwanda as a case study. The following three research questions were investigated: (1) What factors could explain the changes in individual perceptions and malaria-related behaviour over time? (2) What factors could explain the differences and similarities in perceptions and malaria-related behaviour between volunteers and non-volunteers? (3) How do volunteers and non-volunteers benefit from a citizen science programme? The answers to these questions are important to guide the design and implementation of future CSPs and inform policymakers why citizens’ engagement in malaria control activities is vital for malaria elimination.

**Fig. 1:**
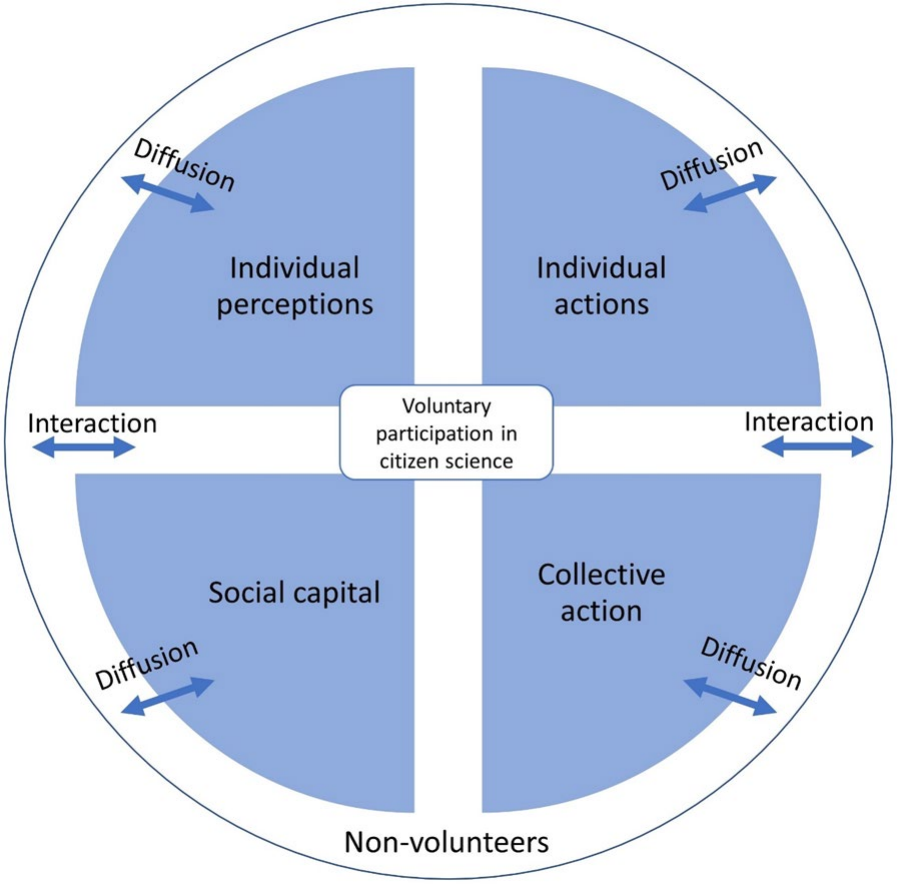
Effect of a citizen science programme among volunteers and non-volunteers at both individual and community levels. Interaction refers to sharing information between volunteers and non-volunteers (those who are not directly involved in reporting of observations), while diffusion indicates the spreading of individual and collections actions

An integrated model of determinants of malaria preventive behaviour was used [19]. This model proposes that engagement in CSPs influences individual perceptions, social capital, and both individual and collective action (see Fig. 1). Volunteers interact and share malaria-related information to non-volunteers, in turn, the impact diffuses to non-volunteers as well. Social capital here refers to interaction and discussion between volunteers and non-volunteers in the neighbourhood or the community, and collective action refers to participation in malaria control activities at the community level.

## Methods

### Study setting and project description

**Fig. 2:**
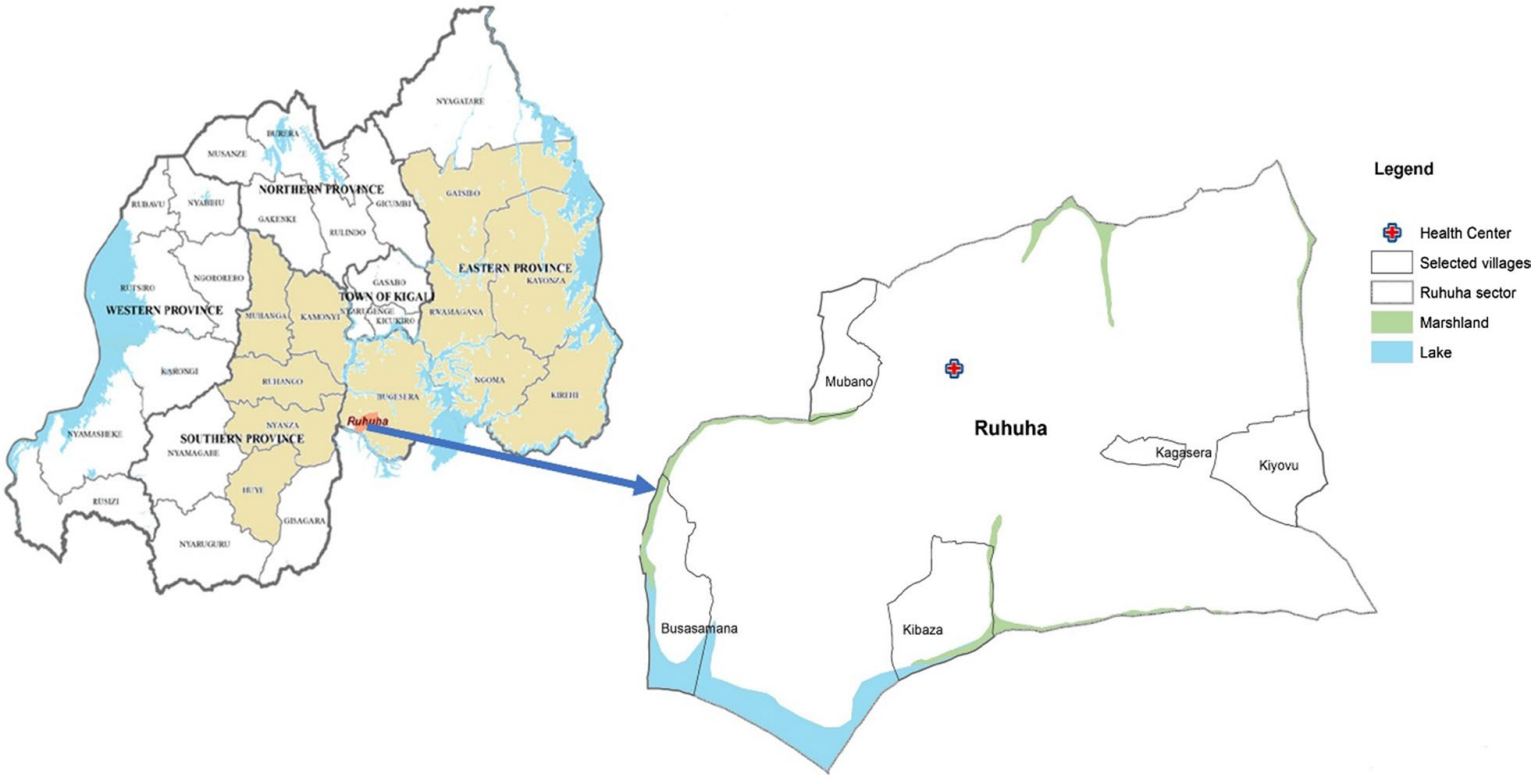
Map of Rwanda indicating Ruhuha sector as a study site and five villages where the citizen science programme was implemented

This study was situated in the Ruhuha sector of the Bugesera district in the eastern province of Rwanda as this region carries a high malaria prevalence relative to other provinces (see Fig. 2). Ruhuha was also selected for the implementation of this CSP because it is a location that does not have a sentinel malaria mosquito surveillance site implemented by the National Malaria Control Programme (NMCP) of Rwanda. The details of the recruitment and design of the programme have been published in an earlier paper [11]. The CSP was implemented to provide data on mosquito density, mosquito nuisance, and malaria cases, to complement the ongoing active surveillance in 12 sentinel sites in the country, and may help NMCP to plan and implement targeted malaria control interventions.

### Study design and population

In this study, a pre- and post-intervention design with mixed methods was used. Two dissemination workshops, five village meetings, and surveys (baseline and end-line) were conducted. All volunteers (112) (see reference for how these were selected) [11] that participated in the citizen science programme were invited to attend two dissemination workshops. Almost all (108 in the first, and 112 in the second workshop) attended. Five village meetings were scheduled and were attended by the respective volunteers. All meetings were conducted at the volunteers’ respective villages as it was a convenient location for them.

**Fig. 3:**
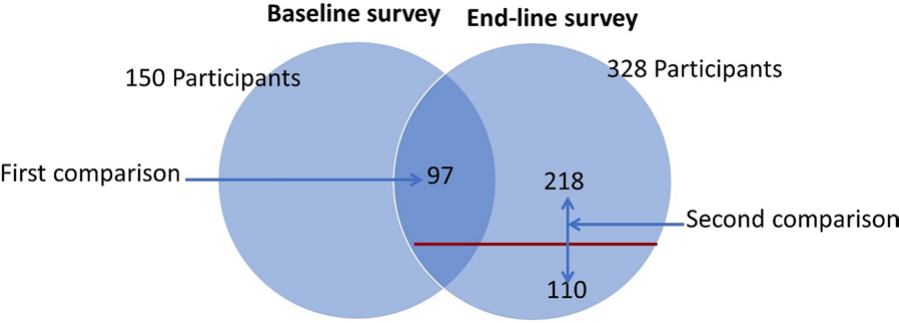
Study population and comparisons that were conducted. The first comparison indicates a pre-post, while the second one is between volunteers and non-volunteers

Baseline and end-line surveys were carried out to conduct two comparisons (see Fig. 3). The first comparison involved changes in malaria-related perceptions and behaviour over time (between 2017 and 2019). A list of people included in the baseline survey conducted in 2017 [4] was used to select the respondents in the end-line survey. Among 150 participants that were involved in the baseline survey in the study area [4], 97 were also available during the end-line data collection, hence they were included. The second comparison was done between volunteers and non-volunteers and involved 328 randomly selected participants. These included 112 volunteers and a double number (224) of non-volunteers. A volunteer:non-volunteer ratio of 1:2 was considered to be able to have enough sample for comparison and assess the benefits of the CSP among volunteers and non-volunteers. However, during data collection two volunteers were not available, and four of the selected non-volunteers were not available as well. Thus, at the end we had 110 volunteers and 218 non-volunteers which yielded a total of 328 for the end-line survey. Figure 3 shows the two comparisons that were made.

### Data collection and study instruments

**Fig. 4:**
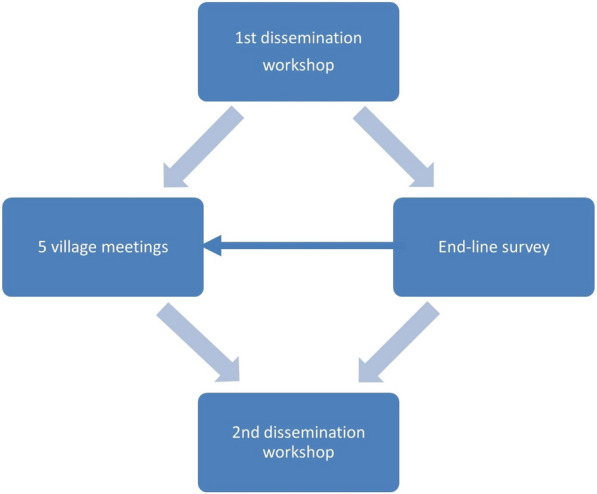
Different data collection methods used and how they follow each other

The data were collected with different methods (dissemination workshops, a survey and village meetings) in three main steps (see Fig. 4).

#### Step 1: first dissemination workshop

Four months after the start of the CSP, a first dissemination workshop was conducted to share the information about what volunteers reported. Four months were chosen based on the agreement and the decision taken during the design phase [11]. Group discussions were held to indicate the benefits of being involved in the programme, what volunteers have learned, and what they have gained through the participation process. To collect this information, a group discussion guide was used.

#### Step 2: end-line survey and village meetings

The second step involved both a survey and five village meetings. The end-line survey was used to compare with the baseline survey [4] and to get information about the benefits of participating in the programme. For case-matched pre- and post-intervention comparison, the same questions that were asked in the baseline survey [4] were repeated in the end-line survey. The main variables included individual perceptions which were measured using a 5 point Likert scale. Individual perceptions included perceived severity (measured with eight statements), perceived susceptibility (measured with seven statements), perceived self-efficacy (measured with seven statements), perceived response efficacy (measured with six statements), perceived barriers (measured with eight statements), norms (measured with nine statements), and behavioural intentions (measured with seven statements). Furthermore, malaria-related behaviour (use of LLINs, indoor residual spraying (IRS), social interaction, and collective action) were measured by one statement each. Based on the outcomes of the first dissemination workshop, closed-ended questions related to the benefits of the CSP were added to the survey questionnaire as well.

Five village meetings were held with volunteers in their villages to discuss the collective action that they may have started and to explore how volunteers share the malaria-related information with non-volunteers. Two main questions guided the meetings: one about the collective action, and another one about sharing malaria information with non-volunteers. They were conducted on the last day of the visit for survey data collection in each village. The main reason to conduct these meetings on the last day was to get insights on the awareness of the programme among non-volunteers. This was received through debriefing sessions with the research assistants at the end of each day.

#### Step 3: second dissemination workshop

The third step involved a second dissemination workshop. It aimed to complement the information about the benefits of being involved in the programme. These included what they have learned, and what they have gained through participation. In addition, the main questions about how volunteers share the malaria-related information to non-volunteers, and what collective action they have initiated as a group, were again discussed in the workshop for validation and sharing of information among all volunteers. The structure of the second workshop was a bit different from the first dissemination workshop in such a way that the discussion groups were mixed (volunteers from different villages). Researchers had to make sure that each village was represented in each group. This was done to ensure that participants could share information with others from different villages. Indeed, this helped the researchers to compare and validate the information shared during village meetings.

### Data analysis

The quantitative data were analysed using SPSS version 25. Composite scores were computed for each variable by calculating the average score for each variable per individual. Non-parametric tests were used for comparisons because the data were not normally distributed. Wilcoxon (matched-pair) signed-rank test was performed to examine the changes in individual perceptions between paired observations (between 2017 and 2019). Furthermore, as the use of malaria-related measures (LLINs and IRS) was measured with one statement each, these were considered as ordinal variables, hence marginal homogeneity tests were used to compare the matched observations (between 2017 and 2019) for these variables.

Further, the Mann–Whitney U test was performed to assess the differences in individual perceptions between volunteers and non-volunteers. In addition, behaviour-related variables were compared using a Chi square test because they were measured with one statement each. Qualitative data were analysed manually to support the quantitative data by providing insight into the underlying reasons for the differences and similarities observed for both individual perceptions and malaria-related behaviour.

## Results

The results are presented in three sections. The first presents the demographic characteristics of the study participants. The second section reports on the changes in malaria-related perceptions and behaviour between 2017 and 2019. In addition, it includes factors that may explain the observed changes. Finally, the third section presents information about the differences and similarities between volunteers and non-volunteers. It also elaborates on the benefits of the CSP for malaria control.

### Participants’ characteristics

Table 1 shows that of the 328 study participants 56.4% were female. Half of the participants had either no education or partial primary school. 57% of the participants were married and most of the participants were farmers (82.3%). The average age of the respondents was 44.1 years (*SD* = 13.6).

**Table 1:** Demographic characteristics of the study participants

Variable	Categories	Frequency	Percentage
Gender	Male	143	43.6
	Female	185	56.4
Education level	None	78	23.8
	Partial primary	97	29.6
	Primary	99	30.2
	Partial secondary	29	8.8
	Secondary	20	6.1
	University	5	1.5
Marital status	Single	16	4.9
	Married	187	57.0
	Cohabited	56	17.1
	Divorced	18	5.5
	Widow	51	15.5
Main occupation	Farmer	270	82.3
	Public servant	7	2.1
	Self-employed	27	8.2
	Private Officer	4	1.2
	Student	1	0.3
	Unemployed	19	5.8
Age	Mean (± SD)	44.1 (± 13.6)	

### Change in malaria-related perceptions and behaviour between 2017 and 2019

The results presented in Table 2 indicated that there were statistically significant changes in perceived susceptibility, perceived self-efficacy, perceived response efficacy, norms, behavioural intentions, and malaria-related behaviour (use of LLINs and IRS acceptance) between 2017 and 2019. There was also a significant decrease in perceived barriers. Perceived severity did not change.

**Table 2:** Changes in malaria-related perceptions and behaviour between 2017 and 2019 (97 paired observations)

Variables	Mean		P-value
	2017	2019	
Perceived severity	4.3	4.4	0.609
Perceived susceptibility	3.0	3.5	< 0.001
Perceived self-efficacy	4.3	4.6	< 0.001
Perceived response efficacy	3.3	4.4	< 0.001
Norms	2.9	3.7	< 0.001
Perceived barriers	2.4	1.5	< 0.001
Behaviour intention	4.5	4.7	0.001
LLINs use	4.0	4.8	0.017
IRS acceptance	4.6	4.9	0.005

Wilcoxon (matched-pair) signed-rank test was used for others while marginal homogeneity tests were performed for Bed net use and IRS acceptance)

The participants were asked whether they noticed any change in their use and acceptance of malaria control measures between 2017 and 2019. In line with the results presented in Table 2, 22% of the participants indicated that they changed the frequency of using bed nets and 51% reported change in IRS acceptance. Regarding the direction of change, among those who reported a change in bed net use and IRS acceptance, 56%, and 95% indicated that the use and acceptance of bed nets and IRS acceptance had increased, respectively (Table 3).

**Table 3:** Change in bed net usage and IRS acceptance

Variables	Categories	Frequency	Percentage
Change in frequency of bed net use	Yes	50	22
	No	173	78
	Total	223	100
	Increased	28	56
	Decreased	22	44
	Total	50	100
Change in IRS acceptance	Yes	166	51
	No	161	49
	Total	327	100
	Increased	158	95
	Decreased	8	5
	Total	166	100

#### Factors explaining the differences observed over time

To interpret the quantitative results presented in Tables 2 and 3, some additional qualitative analyses were conducted. Participants who increased the frequency of bed net use reported to do so because of increased knowledge and awareness about malaria-related benefits of using bed nets, as it prevents people to be in contact with mosquitoes which cause malaria. This indicates the improvement in perceived response efficacy. One participant mentioned:

*“I have increased the frequency of sleeping under a bed net because the bed net prevents me to get into contact with mosquitoes which can cause malaria.”* Another participant stated: *“Because of the workshops and information about malaria that I received, I am now using the bed net every night”.*

For those who mentioned that they have decreased the frequency of sleeping under a bed net, the main reasons highlighted were the perceived decrease of mosquito density, being in a dry season (at the time of data collection), and discomfort (feeling too hot) when sleeping under a bed net. Some respondents said:

“*I have decreased the frequency of sleeping under a bed net because the mosquitoes also have reduced these days.*” Another indicated: “*I have decreased the frequency because now we are in a dry season*.” In relation to discomfort, one mentioned: *“I have decreased the frequency because sometimes it makes me feel too hot.”*

For those who reported no change in frequency of bed net use, most of them indicated that they have been sleeping under a bed net consistently because they want to prevent malaria, or they fear to get malaria, therefore, the frequency of using bed nets remained the same. One participant said:

“*To me, the frequency of sleeping under a bed net was not changed because I have been using it every night so as to prevent malaria.”* Another mentioned, *“I have been sleeping under the bed net every night because I am afraid of getting malaria.”*

Regarding IRS, most of the respondents indicated an increase in acceptance because they became aware and realized that the current insecticide is more effective than what they used to spray. One mentioned:

*“the current insecticide is more powerful than the previous one that they used to spray in our houses”.*

### Individual perceptions and behaviour between volunteers and non-volunteers

A comparison of individual perceptions and malaria-related behaviour between volunteers and non-volunteers was conducted. Table 4 indicates that except for perceived self-efficacy, no statistically significant differences in other individual perceptions could be found between these two groups. However, significant differences were observed in social interaction (discussion about malaria in the community, talking to neighbours about malaria and its control), collective action (participating in malaria-related activities at the community level) the use of LLINs, and IRS acceptance.

**Table 4:** Differences in individual perceptions and malaria-related behaviour between 110 volunteers and 218 non-volunteers

Variables	Mean		P value
	Volunteers	Non volunteers	
Perceived severity	4.4	4.4	0.779
Perceived susceptibility	3.4	3.5	0.302
Perceived self-efficacy	4.7	4.6	0.028
Perceived response efficacy	4.4	4.4	0.960
Norms	3.8	3.7	0.152
Perceived barriers	1.5	1.5	0.114
Behavioral intention	4.8	4.8	0.364
Discussing about malaria in the community	2.9	2.3	< 0.001
Talking to neighbors about malaria and its control	2.9	2.3	< 0.001
Participating in malaria related activities (social/community work)	3.1	2.0	< 0.001
Frequency of using LLINs	4.5	4.7	< 0.001
IRS acceptance	4.9	4.7	< 0.001

Mann–Whitney U test for individual perceptions (first seven variables) and Chi square test for behaviour (last five variables). The mean reported is a mean score at a 5-point Likert Scale based on six-nine statements

#### Benefits of the citizen science programme among volunteers and non-volunteers

The quantitative results presented in Table 4 are supported by the qualitative data collected among volunteers. These data were collected during village meetings and the second dissemination workshop with volunteers. During village meetings, information about how they share malaria-related information with non-volunteers was discussed. In some villages, volunteers started some actions that made the project’s activities visible. For example, in the village of Busasamana, volunteers decided to divide themselves into small groups to conduct visits to the homes of non-volunteers to explain what activities they were doing and to mobilize them for malaria preventive measures. In one village meeting, one volunteer said:

*“We have formed small groups and went into households of non*-*volunteers to show them how they can control malaria. After that, we had a meeting with the whole village, and we demonstrated what we do as volunteers, what they can do to control mosquitoes, and we mobilized them to use malaria control measures in general”.*

The active sharing of information with non-volunteers was also mentioned in the second dissemination workshop, in relation to the question asked during group discussions “How do you share/communicate malaria-related information among non-volunteers in the village”? In response to this question, one group mentioned:

*“When there is a village meeting, we take some time and talk about malaria to inform those who are not volunteers; we occasionally go in their households and mobilize them about malaria prevention. In addition, we sometimes collect mosquitoes in their homes so that they can be aware of what we are doing because some of them ask us to set the trap in their houses”.*

Apart from what volunteers mentioned about sharing the information with non-volunteers, the latter also confirmed this in the end-line survey as reported in the following section. This reports the source of information about the programme, what they learned and gained, as well as indicating their willingness to participate in the programme.

#### Perceptions related to the presence of the citizen science programme in the community

Table 5 shows that 88% of non-volunteers already knew this programme by the time the survey was conducted, and 45% had at least some information about the programme. Among those who had information about the programme, a substantial proportion (73%) received this information from the volunteers. Generally, the majority of both volunteers and non-volunteers judged the programme as very good (68% vs 33%) or good (32% vs 52%), respectively.

**Table 5:** Perception of citizen science programme among 110 volunteers and 218 non-volunteers

Variables	Categories	Frequency	Percent
Heard about the citizen science initiative in this area	No	27	12
	Yes	191	88
	Total	218	100
Informed about the citizen science programme	Not informed at all	120	55
	Not informed	37	17
	Somewhat informed	44	20
	Well informed	15	7
	Very well informed	2	1
	Total	218	100
Source of information	Workshop	5	5
	Volunteer	72	73
	Collected mosquitoes in my house	21	21
	Total	98	100
Having a citizen science programme in the area near your home (volunteers)	Good	35	32
	Very good	75	68
	Total	110	100
Having a citizen science programme in the area near your home (non-volunteers)	Bad	5	2
	Not good and not bad	28	13
	Good	113	52
	Very good	72	33
	Total	218	100
The extent of learning from this citizen science programme (volunteers)	Moderate	13	12
	Much	61	55
	Very much	36	33
	Total	110	100
The extent of learning from this citizen science programme (non-volunteers)	Nothing	133	61
	Little	54	25
	Moderate	22	10
	Much	8	3.5
	Very much	1	0.5
	Total	218	100

#### Learning and gaining from the citizen science programme

**Fig. 5:**
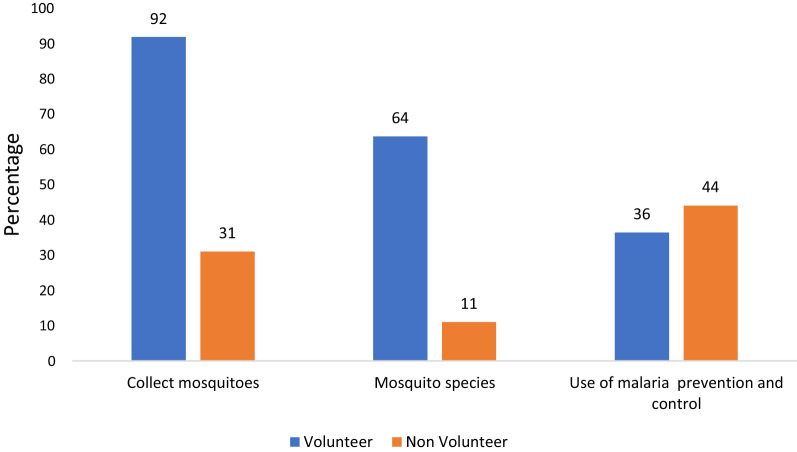
Proportions of volunteers and non-volunteers reporting about what they learned since they had the CSP in the area near their homes (N _volunteers_ = 110; N _non-volunteers_ = 85)

The analysis of what learned and gained was limited to those who heard about the initiative and at least have learned something (from little to very much, i.e. 110 volunteers and 85 non-volunteers see Table 5). Figure 5 indicates that both volunteers and non-volunteers learned some topics from the citizen science programme. Volunteers learned more about collecting mosquitoes (92%) and different mosquito species (64%), while non-volunteers learned more about the use of malaria preventive and control measures (44%).

**Fig. 6:**
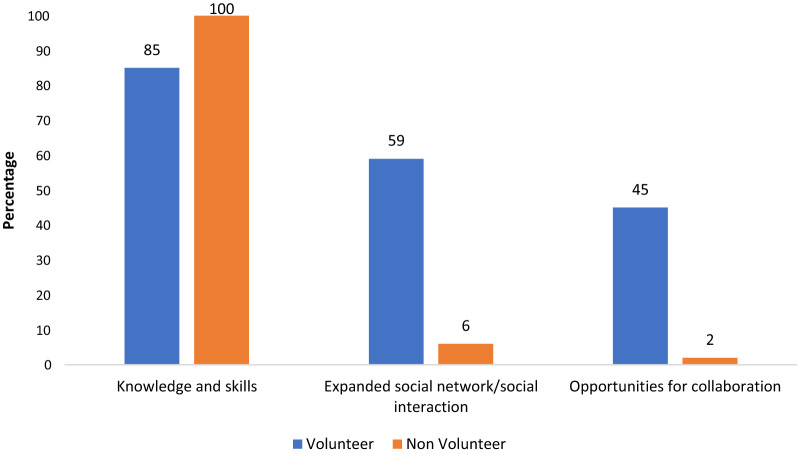
Proportions of volunteers and non-volunteers reporting on what people gained as a result of the CSP (N_volunteers_ = 110; N_non-volunteers_ = 85)

As indicated by Fig. 6, both volunteers and non-volunteers gained knowledge and skills. Additionally, volunteers expanded their social network and gained opportunities for collaboration with peers and researchers.

#### Willingness to join/continue participate in the citizen science programme

**Fig. 7:**
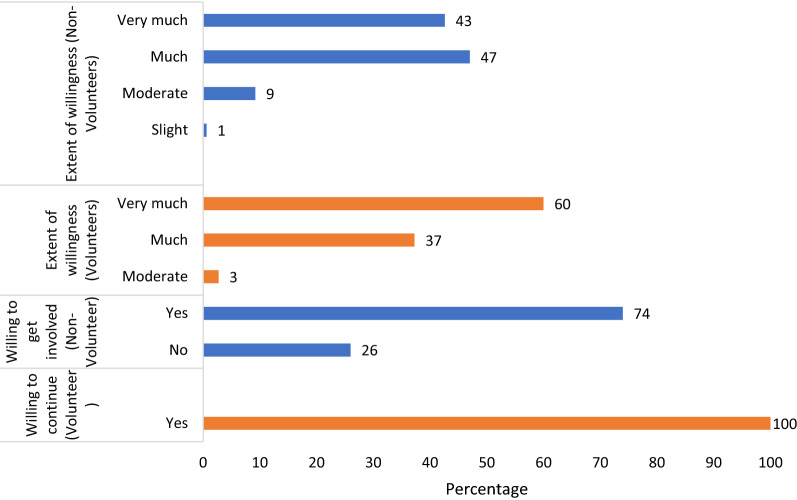
Proportions of volunteers and non-volunteers reporting their willingness to participate in the CSP

Figure 7 indicates that all volunteers were willing to continue participate in the project even after the completion of the research, and a large proportion (75%) of non-volunteers wished to join the project as well.

## Discussion

This study provided quantitative and qualitative insight in the impact of the CSP for malaria control that was conducted in Rwanda. Specifically, it determined the changes in individual perceptions and malaria-related behaviour over time, identified the differences and similarities in perceptions and malaria-related behaviour between volunteers and non-volunteers of a CSP, and explored the reported benefits of the programme.

### Change in perceptions and behaviour

Generally, the research findings indicate that participation in the CSP for malaria control influenced both individual perceptions and malaria-related behaviour among volunteers and non-volunteers. Malaria related perceptions and behaviour increased significantly over time (between 2017 and 2019). When the results were compared between volunteers and non-volunteers, no significant differences in individual perceptions between these two groups could be found. However, significant differences were observed in social interaction, participation in malaria-related activities and IRS acceptance. Significant increases in malaria-related behaviour observed among volunteers in this study corroborate with the literature that indicates that the more people engage in citizen science, the greater the impact on their behaviour [17, 18]. For example, Roetman, Tindle [17] found that through participation in a pets’ related CSP, volunteers who were engaged in the management of their cats reported to change their behaviour and kept their cats indoors more often. Equally, a health promotion related to CSP conducted in The Netherlands also reported intentions and actual changes in lifestyle behaviour among citizen scientists [12].

As a result of participation in CSP, the volunteers have played a large role in the transfer of the malaria-related information to non-volunteers, and this resulted in similarities in individual perceptions reported in this study. The non-volunteers could learn from and be influenced by citizen science projects in many ways [17, 18]. For example, the non-volunteers in the Cat Tracker citizen science project reported changes in attitudes [17]. These changes may be a result of following the project as observers, or interest the non-volunteers may have had in the feedback provided to volunteers by scientists [17]. This sharing of information is analogous to what Rogers [20] coined “diffusion”, a process by which an innovation (for example CSP for malaria control) is communicated among members of a social system over time using different channels to reach a common understanding [20].

As reported in this study, some volunteers planned home visits and meetings at the village level with non-volunteers and explained the project’s activities. In turn, this might have influenced the changes in perceptions and behaviour reported in this study. In line with this, sharing generally positive feedback about the goals and benefits of the project to the wider community by the citizen scientists, and direct discussion about the project with non-volunteers have been reported in citizen science literature [16, 17, 21]. In line with sharing what volunteers learned from citizen science, Bremer, Haque [14] found that some volunteers reported sharing what they learned in the project with students and colleagues [14]. In turn, this may play a large role in the change of attitudes among non-volunteers, and stimulate willingness to participate in other citizen science projects’ activities, or even in other projects [17]. Through discussion and interaction in the community, collective efforts can be made to solve common problems (malaria burden in this case), hence improve the health and wellbeing of the community. In this study, by initiating the discussion about malaria and related control measures, volunteers felt concerned about malaria and felt that the discussion can be the first step towards collective efforts in controlling malaria.

Apart from the discussion about malaria-related activities with neighbours, or with community members in general, other organizational activities have been observed. For example, in this project, volunteers in one village decided to form a cooperative which would facilitate them to meet more often and discuss about malaria and its control. To encourage this, in the cooperative, they decided to contribute a monthly fee and they could rotate among themselves by either buying a domestic animal for each member on a monthly basis or in case a person is not interested in buying domestic animals, then, they could give him/her money to accommodate the needs. In the latter, other members could make sure that the money will not be misused. This indicated that participation in this CSP for malaria control may not only be related to malaria control but may also induce other financial gains. When time continues, there is no hesitation that this may also be open to non-volunteers who might be interested. Similarly, in other CSPs, some additional activities, and interesting cases were reported. For example in a cat tracker CSP conducted in South Australia [17], one volunteer engaged neighbours to volunteer their pets for tracking and organized different meetings to discuss the results. Beyond the discussion, the same author added that the volunteer had more curiosity related to animal behaviour and returned to school to study the related subject [17].

Participation in malaria-related activities, social and community work (for example to clear mosquito breeding sites) was higher among volunteers. In the same way, an increase of participation in conservation activities, more engagement in CSP-related activities, as well as joining other projects at national and regional levels have been reported in a CSP on coastal observation and seabird survey conducted in South Australia [16]. Joining other invasive plant removal projects and changes in planting habits have been also reported by volunteers in a CSP related to the ecology of invasive plants [21].

### Learning in a citizen science programme

In the present study, both volunteers and non-volunteers reported that they have learned about how to collect mosquitoes, identify different mosquito species, and use malaria control measures. Consistently, learning new things and awareness raising especially about the object of study has been reported by many researchers in CSPs [16, 18, 22–24]. For example, a CSP conducted in the north of the Philippines about ecosystem functions reported an increase in awareness about different species that they were collecting [22]. Likewise, Den Broeder et al. [12] also reported an increase in social skills and self-confidence in talking and discussing with non-volunteers about health-related topics such as healthy lifestyle and health promotion activities. The reported knowledge and skills gained in the current study may be helpful as the citizens reported the lack of information about mosquitoes during the design phase of the project [11], and were interested in discussing control of mosquito breeding sites [11]. Thus, the knowledge and skills gained may help them in the control of mosquito breeding sites in the area.

Apart from the knowledge and skills gained among both groups, volunteers also reported having expanded their social network, and opportunities for collaboration. By participating in citizen science, volunteers meet new people, interact with them and become friends with several people in volunteers’ groups, neighbourhood, and beyond [12, 14]. For example, in a CSP on climate adaptation conducted in Bangladesh volunteers created a network (which they referred to as a family) that helped them to interact regularly outside of the project’s meetings [14]. These social networks and interactions often enhance trust and social cohesion among people in the community [12, 14].

### Willingness to become involved or continue participating in the citizen science programme

Willingness to become involved, or continue participating in the programme was assessed and the majority (100% of volunteers and 75% of non-volunteers) indicated that they were. In the evaluation of citizen science projects, other researchers also assessed the willingness to participate in a new project. For example, in their study about koala management, Hollow et al. [18] revealed that 91% of the volunteers, 54% of those who heard about the project, and 29% of those who are not informed about the project were willing to participate in another koala related project. In the same line, in a water quality citizen science project, Brouwer and Hessels [24] also revealed a high level (90%) of willingness to participate in future studies related to water quality measurements. The high level of willingness reported in the present study may reveal the concern of the citizens about the malaria burden, and probably their interest for participation in related scientific research.

### Implications of the findings to the citizen science practice

When implemented, CSPs may have different objectives [24]. Most of the co-designed CSPs target scientific, societal, and political impacts. In this regard, an integration of these elements at the start of the project, and establishing how they will be assessed and achieved is key to realize the full potential of CSPs. Following the findings reported in this study, some key elements related to societal impact merit attention and inclusion in the citizen science evaluation framework. These include the nature of learning and learning arrangements. The latter may be thoroughly planned before the implementation of CSPs. This is because in most cases, CSPs are mainly considered as a tool to facilitate collection of environmental data, and most scientists give priority to the quality and quantity of citizen science data, but not to the educational part of the initiative. However, the educational aspect of citizen science may influence the quantity and quality of data. Therefore, pairing ecological data (scientific impact) and social data (societal impact) may provide tangible evidence to policymakers for political impact.

While the effect of citizen science extends beyond those who are actively participating, many CSPs only assess learning and consider this as the main outcome in citizen science [25]. This is mainly because of a lack of pre-determined criteria to be assessed throughout the project’s period. This study provides evidence that CSPs offer other benefits including environmental management at the community level. Therefore, researchers in citizen science should explore the benefits of citizen science beyond volunteers at both individual and collective levels. In citizen science, long term engagement may be more influenced by the societal impact [26]. This is to say that co-designed CSPs that are based on existing societal problems (for example malaria in this case) or build upon a joint interest between researchers and citizen scientists may lead to successful participation and achieve the educational goal [25]. This implies that the evaluation of CSPs should take into consideration the nature of the project and the design used.

### Strength, limitation and further research

Overall, the findings reported in this study merit consideration in future CSPs, as they contribute to the design and implementation, as well as the sustainability of CSPs. For example, the comparison made between volunteers and non-volunteers provides useful information related to the educational and citizen engagement goals of the project.

Most of the respondents who reported change in perceptions and behavior reported doing so because they have acquired information related to the use of malaria control measures, and this information may be partially attributed to the CSP for malaria control. The change of perceptions and behaviour over time cannot be associated completely with the programme because quantification of the effect of the CSP on volunteers (pre-post interventions for the volunteers only) was not done. On the other hand, given that there was no any other malaria initiative in the area during the study period, the substantial impact may be attributed to this CSP. Further studies quantifying the impact of this programme with a comparison of some community members away from the study site (for example Busoro sector which was involved in the baseline study [4]) as a control group is desired.

The current study reported outcomes at both individual and community levels involving those who are directly engaged in the collection of citizen science data, and those who are not directly involved. However, some outcomes including community leadership, organizational capacity to address collective problems, and improved community well-being were not measured. Therefore, as a citizen science programme may have a capacity to improve these outcomes, future studies should include such key variables as well.

The citizen science volunteers collected and reported citizen science data (mosquito species, mosquito nuisance, and confirmed malaria cases) on a monthly basis for 1 year. However, the current study only reported on the societal impact of this programme. Other studies reporting the scientific impact (citizen science data submitted by the volunteers) of the current programme should also be conducted.

Finally, some studies have demonstrated that CSPs play a large role in the policy arena [18]. This societal impact of the CSP for malaria control presented in this study provides evidence that it is useful for decision-makers and policy development for malaria elimination. Further studies that involve policy-makers are needed to determine their perceptions of the programme, and how it can complement the active surveillance of the national malaria control programme.

## Conclusion

This study assessed the quantitative and qualitative impact of a CSP for malaria control. The study offers empirical evidence of the extent to which and how a CSP improves perceptions and use of malaria control measures. From the research findings, it was observed that the individual perceptions in general and malaria-related behaviour improved significantly over time (between 2017 and 2019), thereby becoming more favourable to malaria control. When the results were compared between volunteers and non-volunteers, a significant difference was observed only for the perceived self-efficacy. However, it was apparent that in general, volunteers perform malaria-related behaviour more than non-volunteers. Volunteers and non-volunteers reported gaining knowledge and skills about the use of malaria control measures in general, and mosquito species in particular among volunteers. In fact, the use of LLINs was more among non-volunteers than volunteers. Indeed this shows the diffusion of CSP-related information in the community and gives promise that the non-volunteers may also adopt other malaria-related behaviours similar to the volunteers. Thus, a CSP has potential not only as a means of collecting a large amount of citizen science data, but also equally important, as a means of engaging citizens in decision-making and solving environmental and public health problems.

## Data Availability

The datasets used in this study are available from the corresponding author on a reasonable request.

## Abbreviations

CSP: Citizen Science Programme
IRS: Indoor residual spraying
LLINs: Long-lasting insecticidal nets
NMCP: National Malaria Control Programme
SMS: Short Message Service
